# The expression of Mas-receptor of the renin–angiotensin system in the human eye

**DOI:** 10.1007/s00417-015-2952-z

**Published:** 2015-02-14

**Authors:** A. Vaajanen, G. Kalesnykas, H. Vapaatalo, H. Uusitalo

**Affiliations:** 1 Department of Ophthalmology, Tampere University Hospital, P.O. Box 2000, 33521 Tampere, Finland; 2 SILK, Department of Ophthalmology, School of Medicine, University of Tampere, 33014 Tampere, Finland; 3 Experimentica Ltd., Microkatu 1, P.O.Box 1188, 70211 Kuopio, Finland; 4 Institute of Biomedicine, Pharmacology, University of Helsinki, P.O. Box 63, 00014 Helsinki, Finland

**Keywords:** Mas- receptor, Ang(1–7), Ang II, RAS, Immunohistochemistry, Glaucoma, Human eye

## Abstract

**Purpose:**

The local renin–angiotensin system has been held to be expressed in many organs, including the eye. It has an important role in the regulation of local fluid homeostasis, cell proliferation, fibrosis, and vascular tone. Mas-receptor (Mas-R) is a potential receptor acting mainly opposite to the well-known angiotensin II receptor type 1. The aim of this study was to determine if Mas-R is expressed in the human eye.

**Methods:**

Seven enucleated human eyes were used in immunohistochemical detection of Mas-R and its endogenous ligand angiotensin (1–7) [Ang(1–7)]. Both light microscopy and immunofluorescent detection methods were used. A human kidney preparation sample was used as control.

**Results:**

The Mas-R was found to have nuclear localization, and localized in the retinal nuclear layers and in the structures of the anterior segment of the eye. A cytoplasmic immunostaining pattern of Ang(1–7) was found in the inner and outer nuclear and plexiform layers of the retina and in the ciliary body.

**Conclusion:**

To the best of our knowledge, this is the first report showing Mas-R expression in the human eye. Its localization suggests that it may have a role in physiological and pathological processes in the anterior part of the eye and in the retina.

## Introduction

**Fig. 1:**
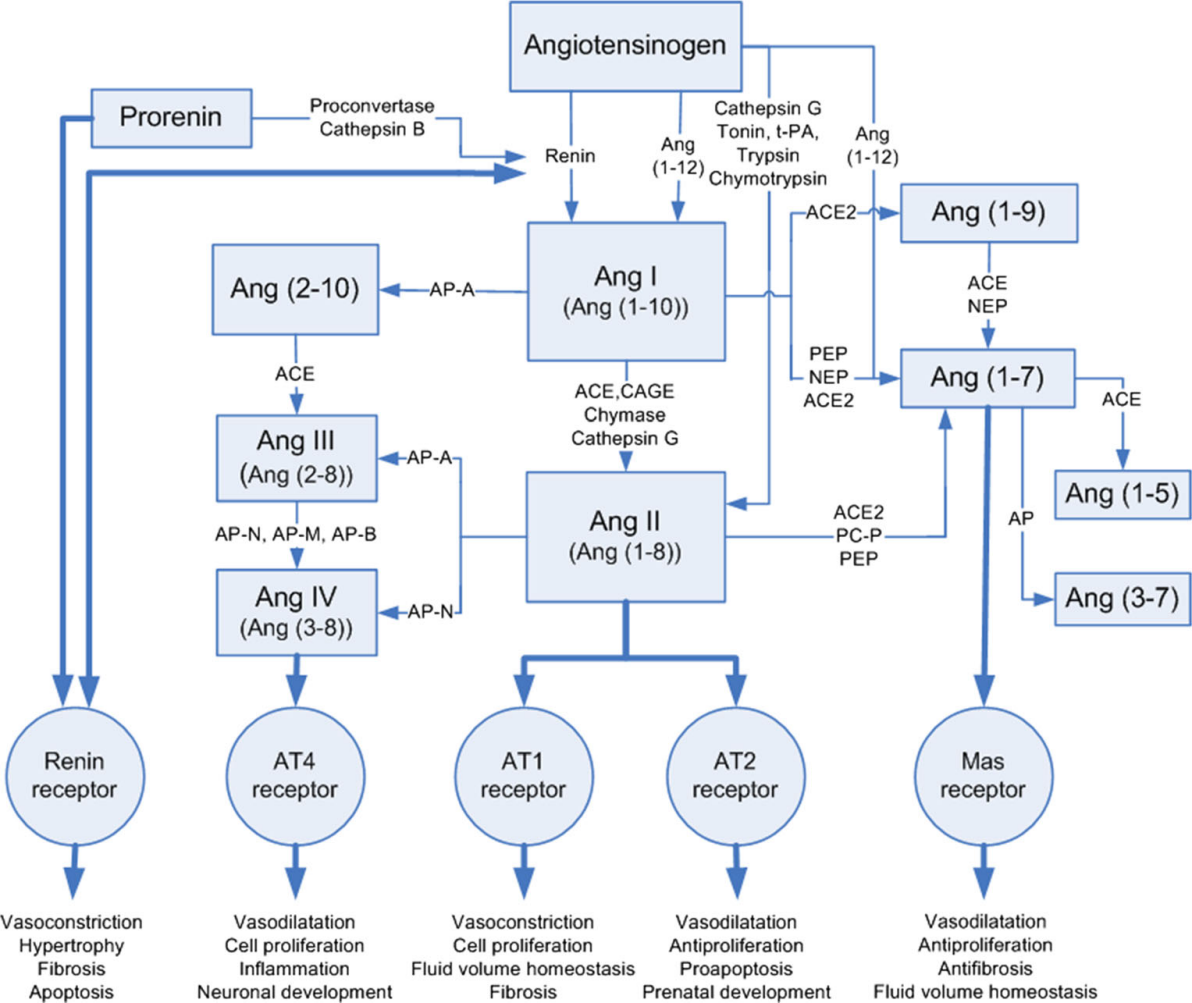
A RAS cascade. *ACE* = angiotensin-converting enzyme, *ACE2* = angiotensin-converting enzyme-related carboxypeptidase, *Ang I,II,III,IV* = angiotensin I,II,III,IV, *Ang (1–10)* = angiotensin (1–10), *Ang (1–8) *= angiotensin (1–8), *Ang (2–8)* = angiotensin (2–8), *Ang (3–8) *= angiotensin (3–8), *Ang (1–9) *= angiotensin (1–9), *Ang (1–7) *= angiotensin (1–7), *Ang (1–5) *= angiotensin (1–5), *Ang (3–7) *= angiotensin (3–7), *AT1 *= angiotensin II type 1 receptor, *AT2* = angiotensin II type 2 receptor, *AT4* = angiotensin II type 4 receptor, *AP* = aminopeptidase (−A,-N,-M,-B), *CAGE *= chymostatin-sensitive Ang II-generating enzyme, *Mas-receptor *= Ang (1–7) receptor type, *Nep* = neprilysin, *PEP* = prolyl endopeptidase, *PC-P* = prolylcarboxy-peptidase, *tPA* = tissue-type plasminogen activator. (Vaajanen et al. 2008a, a modified version)

The systemic renin–angiotensin system (RAS) regulates body fluid balance, blood pressure, and many cardiovascular responses via endocrine mechanisms. Vasoconstrictive angiotensin II (Ang II) has a key role in RAS by acting mainly via the angiotensin type 1 receptor (AT1-R) [1, 2]. In addition to the classical AT1-R and angiotensin type 2 (AT2-R) receptors, another angiotensin receptor type, a Mas-receptor (Mas-R), has been identified [3, 4]. Many of the Mas-R effects are opposite to those of AT1-R. Both have important but opposite roles in the regulation of local fluid homeostasis, cell proliferation, fibrosis, and vasodilatation. Mas-R is activated by an endogenous heptapeptide, angiotensin 1–7 [Ang (1–7)], which in turn is a degradation product of octapeptide Ang II (see Fig. 1 illustrating the RAS cascade). In addition to the systemic RAS, a local tissue RAS has been identified in which long-term changes are regulated by autocrine and paracrine mechanisms [5]. Local RAS has been widely described in the heart, kidney, and adrenal tissue, but also in other organs such as the brain, reproductive system, intestine [5–7], and the eye [8–14]. The present study was designed to investigate localization of Mas-R and Ang(1–7) in the human eye using immunohistochemistry.

## Materials and methods

### Eyes and fine-needle samples of the kidney

The study was approved by the local ethical committee (Regional Ethics Committee of the Expert Responsibility Area of Tampere University Hospital, R10155). Seven human eyes were used for detection of Mas-R and its endogenous ligand Ang(1–7). The eyes had earlier been enucleated due to ocular malignancies (all melanomas) at the Tampere University Hospital Eye Center, Tampere, Finland, and were stored in paraffin blocks after removal. As control tissue we used human kidney fine-needle samples originally taken at the Tampere University Hospital for other purposes. The kidney samples were stored in paraffin as well.

### Immunohistochemistry

We used antibodies against Ang(1–7) Mas receptor (AAR 013, Alomone Labs Ltd, Jerusalem, Israel) and Ang-I(1–7)/AngII(1–7) (H-002-24, Phoenix Pharmaceuticals, Inc., Burlingame, CA, USA). The antigen reactivity of both agents has been confirmed for humans but also for the rat (data not shown). For immunohistochemistry we used 4 μm-thick paraffin sections that were cut onto SuperFrost plus microscopic slides (Menzel-Gläser, Gerhard Menzel GmbH, Braunschweig, Germany). The sections were dried at +60 °C for 1 h. Antigen retrieval was performed on re-hydrated sections in a microwave oven at 850 W twice for 7 min using 10 mM Tris 1 mM EDTA retrieval buffer (pH 9.0) as retrieval solution.

### Light microscopy

Endogenous peroxidase activity was blocked with phosphate-buffered saline (PBS) and H_2_O_2_ solution (0.3 %) for 30 min in determination of Mas-R and real peroxidase blocking solution (S2023, DAKO, Glostrup, Denmark) for 5 min in determination of Ang(1–7); thereafter, the samples were rinsed with PBS containing Tween 20. Normal goat serum (50062Z, Invitrogen, Life Technologies, Carlsbad, CA, USA) was used to prevent non-specific staining for 30 min, and then blotted off. AAR 013 was diluted (1:300) in PBS (pH 7.2) containing 0.1 % bovine serum albumin (BSA, A-4503, Sigma, Saint Louis, MO, USA) and incubated for 30 min at +37 °C. H-002-24 was diluted (1:200) in DAKO antibody diluent (S2022, DAKO) at pH 7.2 and incubated for 1 hour at room temperature (RT). Immunostaining was carried out using goat anti-rabbit Ig HRP (1:200, P0448, DAKO) for 30 min as secondary antibody for AAR 013, and Nischirei Histofine (Nischirei Biosciences Inc., Tokyo, Japan) for 30 min for H-002-24. Separate Nischirei Histofine was used for human (414141 F) and rat (434181 F) samples. Aminoethylcarbazole (Vector Laboratories, Burlingame, CA, USA) was used as chromogen and hematoxylin (Merck KGaA, Darmstadt, Germany) as counterstain.

### Fluorescent microscopy

After antigen retrieval, the sections were washed with PBS containing Tween 20 and primary antibodies AAR 013 (1:300) and H-002-24 (1:200) were diluted in DAKO antibody diluent (S2022, DAKO) and incubated overnight at RT. Then the sections were rinsed with PBS-Tween 20 and goat anti-rabbit Alexa Fluor 488 (1:500, Invitrogen, Life Technologies) were applied for 3 hours at RT. In order to confirm the nuclear localization of Mas-R, we double-stained sections with mouse anti-human NCL-Emerin (1:20, clone 4G5, Leica Biosystems Newcastle Ltd., Newcastle Upon Tyne, UK), which specifically labels the nuclear envelope. The cellular-specific presence of Mas-R and Ang(1–7) was investigated by employing antibodies directed against neuronal nuclei (NeuN, 1:100, MAB377, EMD Millipore, Billerica, MA, USA), glial fibrillary acidic protein (GFAP, 1:200, clone 1B4, BD Pharmingen, BD Biosciences, San Jose, CA, USA) that labels retinal astrocytes and activated Müller cells, and endothelial cell marker CD31 (1:100, ab28364, Abcam, Cambridge, MA, USA). Second primary antibodies were diluted in DAKO antibody diluent (S2022, DAKO) and incubated overnight at RT. Then the sections were rinsed with PBS-Tween 20, and goat anti-mouse Alexa Fluor 594 IgG (1:500, Invitrogen, Life Technologies) were applied for 3 hours at RT. The sections were coverslipped using Vectashield mounting medium with DAPI (H-1200, Vector Laboratories, Inc., Burlingame, CA, USA). The sections were analyzed using Zeiss LSM 700 confocal microscope (Carl Zeiss, Germany).

### Negative controls

The sections were stained both by omitting primary and by omitting secondary antibody. In addition, the pre-adsorption control where antibody against Mas-R was pre-adsorbed with antigenic peptide was used. No corresponding staining was observed in any of the negative control samples.

## Results

### Expression of Mas-receptors

**Fig. 2:**
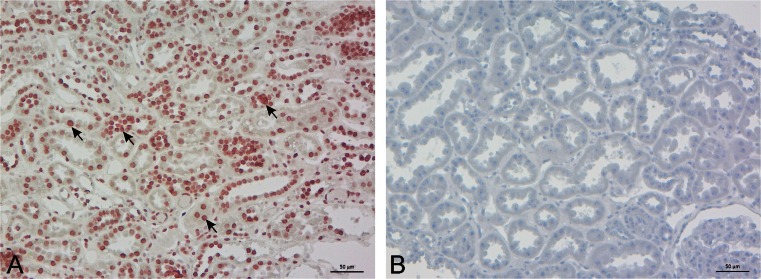
Expression of Mas-receptor in the human kidney. **a** Mas-R (in *red*, *black arrows*) is especially found in the cells of glomerulus, tubulus and vascular endothelium. **b** No corresponding staining was observed in the negative control sample by omission of primary antibodies. Magnification 200×

Mas-R is well known to be expressed in the kidney, adrenals, and vascular system cells [4–6] . Therefore, we chose human kidney tissue as positive control for the antibody recognizing Mas-R. In the kidney tissue, Mas-R was mainly expressed in the tubular and glomerular cells and vascular endothelium (Fig. 2).

**Fig. 3:**
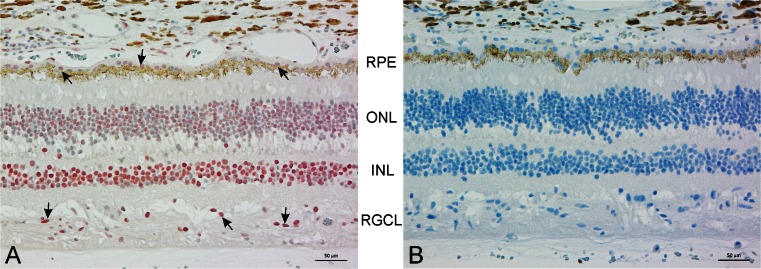
Expression of Mas-R in the human retina. **a** The intensive Mas-R staining (in *red*) was seen in the inner nuclear layer, where most of the cells expressed Mas-R protein. In addition, there were positive cells in the outer nuclear layer of the retina and in the ganglion cell layer cells and retinal pigment epithelium cells. *Black arrows* indicate some staining marks of the Mas-R. **b** No Mas-R immunoreactivity was detected in the negative control sample, which was counterstained (in *blue*) to reveal retinal layers. Magnification 200×. *RPE* = retinal pigment epithelium, *ONL* = outer nuclear layer, *INR* = inner nuclear layer, *RGCL* = retinal ganglion cell layer

**Fig. 4:**
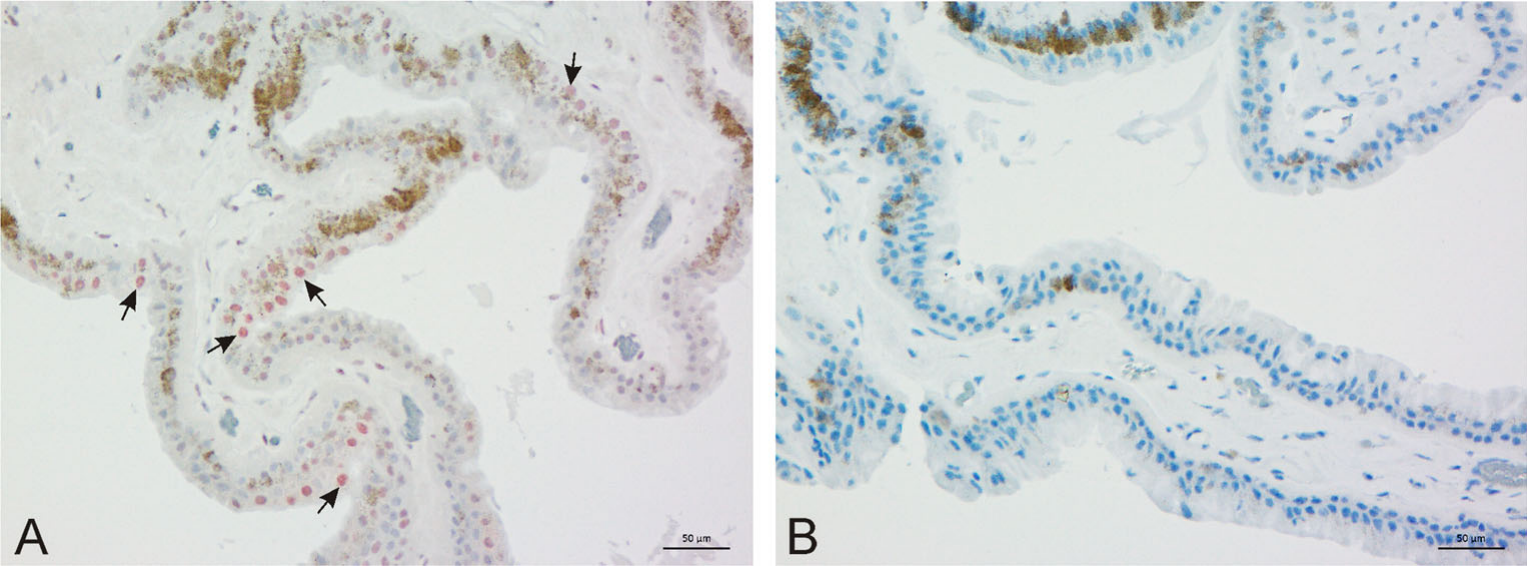
Expression of Mas-R in the human ciliary body. **a** In ciliary body, Mas-R expression (in *red*) was high in non-pigmented cells of the ciliary epithelium. The pigment epithelium cells were also strongly stained. In addition, some cells in the trabecular meshwork and in the wall of Schlemm’s canal were stained (not illustrated in figures). **b** No Mas-R immunoreactivity was detected in the negative control sample, which was counterstained (in *blue*) to reveal retinal layers. Magnification 200×

**Fig. 5:**
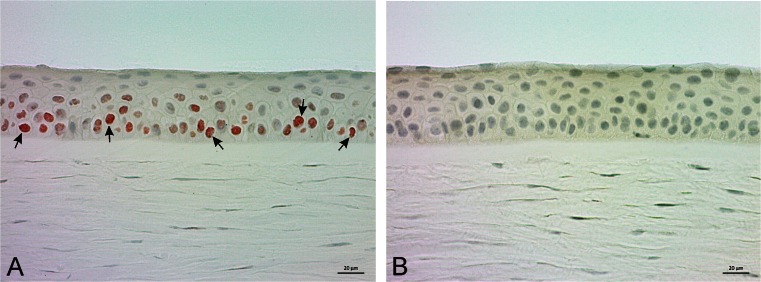
Immunohistochemical staining of Mas-R in the human cornea. **a** Many of the basal and superobasal epithelial cells were stained (in *red*, indicated by *black arrows*). **b** No corresponding staining was observed in the negative control sample. Magnification 200×

Next, we immunostained human eye sections using the same antibody. Mas-R was widely expressed in the eye structures. In the retina, Mas-R was found in the nuclear layers as well as in the retinal pigment epithelium and choroid (Fig. 3). In ciliary body, Mas-R was expressed by non-pigmented cells and the ciliary epithelium (Fig. 4). Mas-R immunoreactivity was also observed in the trabecular meshwork and in the wall of Schlemm’s canal. In the human cornea, the majority of the basal and superobasal epithelial cells were immunopositive for Mas-R (Fig. 5).

**Fig. 6:**
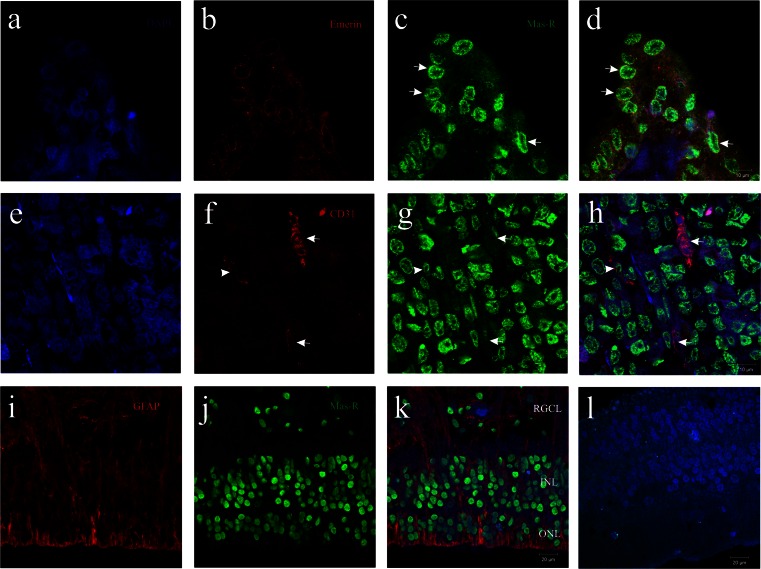
Photomicrographs demonstrating counterstain DAPI (in *blue*), Emerin, CD31 and GFAP (*all in red*) and Mas-R (in *green*). Nuclear localization of Mas-R was found in the ciliary body (**a**–**d**), choroid (**e**–**h**) and retina (**i**–**k**). Pre-adsorption control did not show the corresponding Mas-R immunoreactivity (**l**).*White arrows* in **c** and **d** indicate some cells that colocolized Emerin and Mas-R (in *yellow* in **d**), whereas *white arrows* in **f**–**h** point to CD31-immunoreactive cells of choroid that are immunonegative for Mas-R. However, *white arrowheads* in **f**–**h** indicate endothelial cell that has nuclear Mas-R immunoreactivity. *RGCL* = retinal ganglion cell layer, *INL* = inner nuclear layer, *ONL* = outer nuclear layer

To verify subcellular localization of Mas-R, we used double immunofluorescent staining of sections with antibody directed against Emerin, a nuclear envelope protein. In corneal, ciliary body, retinal, and choroid cells, Mas-R immunoreactivity was exclusively localized in the nucleus of cells (Fig. 6).

### Expression of Ang(1–7)

**Fig. 7:**
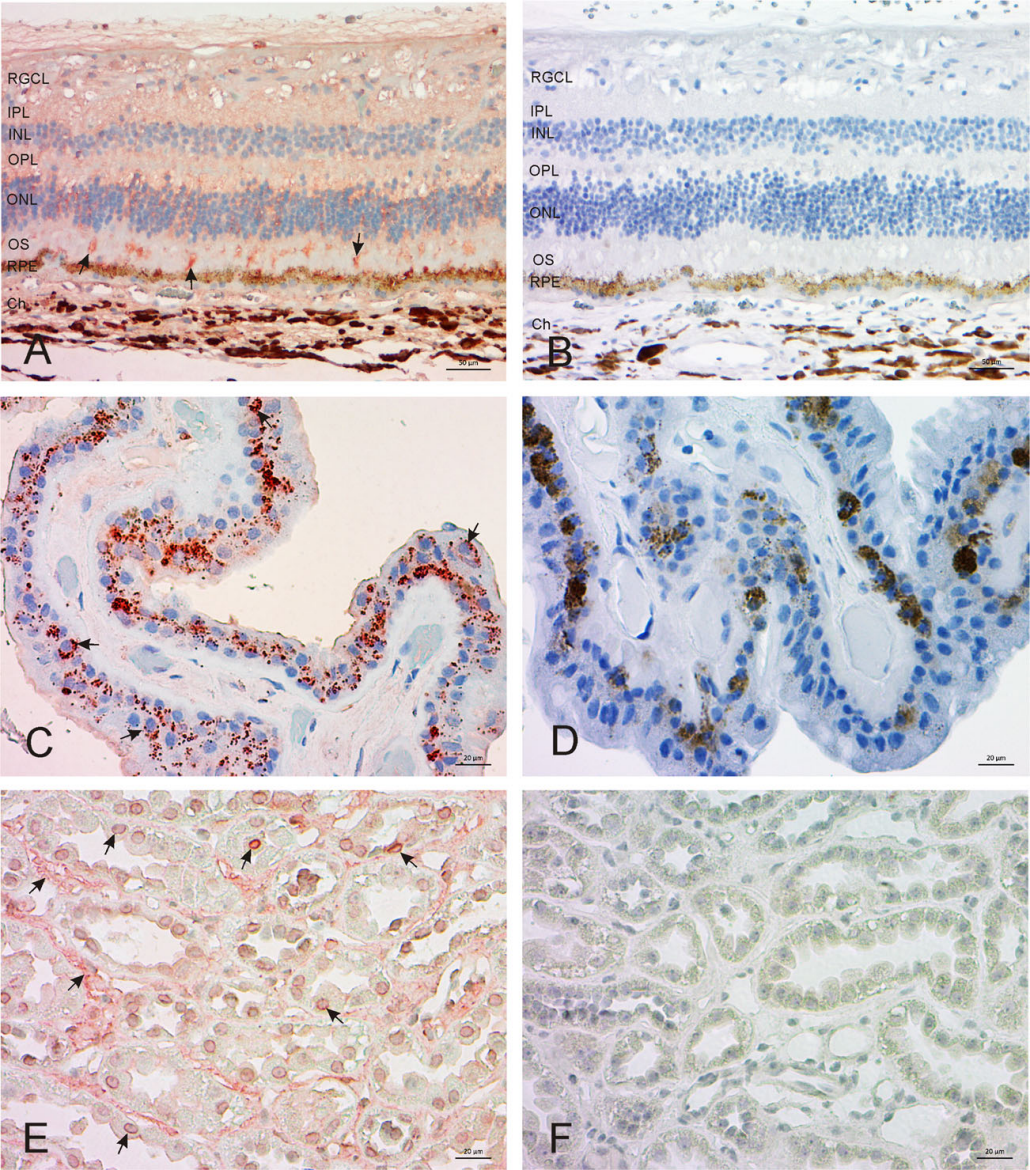
Expression of cytoplasmic Ang(1–7) in the human retina and ciliary body. A weak immunostaining of Ang(1–7) was found in the inner and outer nuclear and plexiform layers of retina **a** In the ciliary body there was a cytoplasmic stain in the non-pigmented and pigmented epithelial cells (**c**). Magnification 200×. A human kidney sample is shown in **e**. Magnification 400×. In all figures, *red–brown* color indicates staining of Ang (1–7), see *black arrows*. **b**, **d**, **f** Show negative staining samples without labelling antibody. *RGCL* = retinal ganglion cell layer, IPL = inner plexiform layer, *INL* = inner nuclear layer, OPL = outer plexiform layer, *ONL * = outer nuclear layer, *OS* = outer segment, *RPE *= retinal pigment epithelium, *Ch* = choroidea

**Fig. 8:**
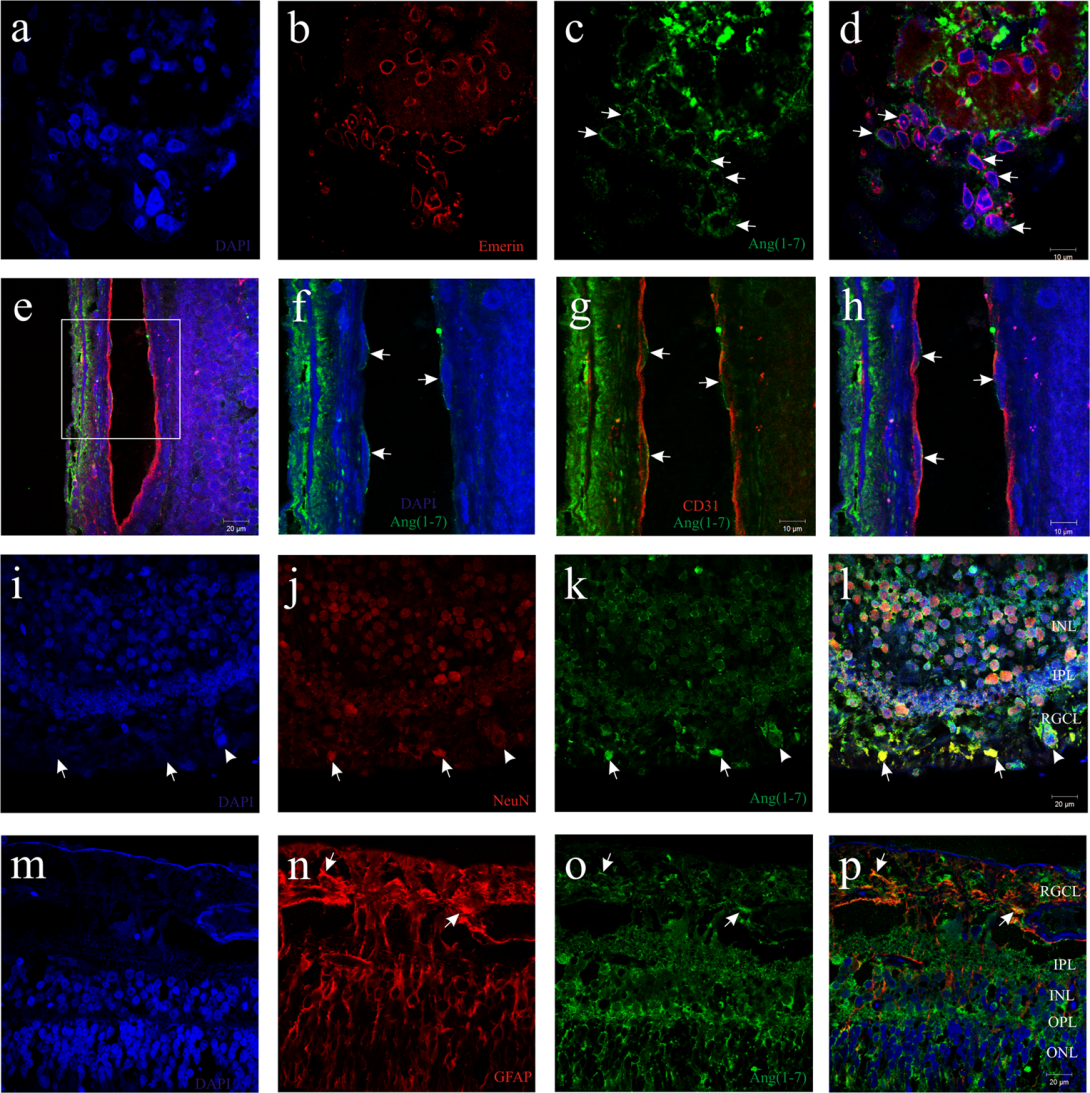
Colocalization of Ang(1–7) (in *green*) with Emerin (*red* in **a**–**d**), CD31 (*red* in **e**–**h**), NeuN (*red* in **i**–**l**) and GFAP (*red* in **m**–**p**). *Blue* color in all photomicrographs indicates counterstain with DAPI. **c** and **d** *White arrows* indicate cytoplasmic localization of Ang(1–7) in ciliary body. **f**–**h** *White arrows* point to CD31-immunoreactive cells localized in the retina and containing Ang(1–7). **i**–**l** *White arrows* indicate NeuN-immunoreactive neurons from the retinal ganglion cell layer, which also contain Ang(1–7) expression. **m**–**p** Retinal glia overexpress GFAP. Some areas (*white arrows* in **n**–**p**) show colocalization of GFAP and Ang(1–7). *RGCL* = retinal ganglion cell layer, *IPL* = inner plexiform layer, INL = inner nuclear layer, *OPL* = outer plexiform layer, *ONL* = outer nuclear layer

Ang(1–7) expression was first verified in the human kidney tissue. Thereafter, Ang(1–7) expression was identified in the human ocular sections. Similarly to Mas-R immunoreactivity, Ang(1–7) was widely expressed in the ocular tissue. We found Ang(1–7) immunoreactivity in the inner and outer nuclear and plexiform layers of retina, choroid, non-pigmented, and pigmented epithelial cells of the ciliary body (Fig. 7). In contrast to Mas-R immunoreactivity, Ang(1–7) was expressed in the cytoplasm of cells and in some cells colocalized with neuronal marker NeuN, glial cell marker GFAP and endothelial cell marker CD31 (Fig. 8).

## Discussion

In this study, we describe Mas-R and its endogenous ligand Ang(1–7) immunoreactivity and cellular localization in human ocular tissue. We found that Mas-R is widely expressed in the eye, with primarily nuclear localization in cells. Ang(1–7) was also found in different structures of the eye, but with cytoplasmic localization in cells. Previously, our research group has described Mas-R expression in the rat eye using the RT-PCR method [15].

Mas-R was first discovered by Young et al. (1986) as proto-oncogene almost three decades ago [3]. Later, the same group reported high Mas-R levels in the rat central nervous system [16]. Two decades ago, Kitaoka and co-workers (1994) demonstrated Mas-R expression in the eyes of rhesus macaque using in-situ hybridization and epi-polarization microscopy [17]. The authors concluded that Mas-R could be used as a specific marker for retinal pigment epithelium cells [17]. However, the functional significance of Mas-R and its putative ligand Ang(1–7) expression in the ocular structures remains unknown. Mas-R is an important member of the local tissue RAS, acting mainly opposite to the well-known AT1-R. Mas-R participates in cell proliferation and antifibrosis as well as vasodilatation and local fluid volume homeostasis. In fact, the potentials of Mas-R ligands, e.g. Ang(1–7) and active enzymes such as ACE2 in degrading vasoconstrictive Ang II to vasodilatory peptides constitute a present focus of cardiovascular drug development [18–20]. Most components of the local RAS have already been widely identified in eye structures [10, 21]. However, further studies are required to identify the specific physiologic role of local RAS in the human eye and possible involvement in such pathological conditions as diabetic retinopathy, age-related macular degeneration, and glaucoma.

In a previous in-vivo study, we found that intraocular administration of Mas-R agonist Ang(1–7) lowers intraocular pressure (IOP) in normotensive rabbit eyes without influence on aqueous humor outflow facility [21]. In human studies, orally administered ACE inhibitor (Captopril) and AT1-R antagonist (Losartan) reduced IOP in both ocular normotensive and glaucomatous subjects [22, 23]. Oculohypotensive effects of ACE inhibitor Perindopril have also been reported in both acute and chronic experimental models of glaucoma [24], as recently also the anti-glaucomatous effects of the activation of intrinsic angiotensin-converting enzyme 2 in an experimental glaucoma [9]. Our data from human eyes indicate that Mas-R is localized in the human non-pigmented epithelial cells of the ciliary body as well as in the cells of trabecular meshwork. The latter suggests that the aqueous humor production and outflow may be influenced by Mas-R and its ligand Ang(1–7). However, further studies are needed to confirm that.

To conclude, expression of RAS components and their cellular localization suggest their involvement in controlling aqueous humour dynamics and intraocular pressure. Furthermore, our descriptive data suggest that RAS may influence pathophysiology of ocular diseases affecting ocular blood flow and vasculature such as diabetic retinopathy. However, further studies are needed to understand the functional significance of the local RAS in the eye.
